# Synthesis of Iron(II,III) Oxide–Titanium Core–Shell Particles via Magnetron Sputtering for Magnetoactive Elastomers

**DOI:** 10.1021/acsomega.5c10485

**Published:** 2026-03-03

**Authors:** Cristian Padilha Fontoura, Amanda Poletto Santi, Wellington Vieira de Souza, Mariana Roesch-Ely, Cesar Aguzzoli

**Affiliations:** † Área do Conhecimento de Ciências Exatas e Engenharias, Graduate Program in Materials Science (PPGMAT), 58802 Universidade de Caxias do Sul, Rua Francisco Getúlio Vargas, 1130, Caxias do Sul, RS 95070-560, Brazil; ‡ Instituto de Biotecnologia, Universidade de Caxias do Sul, Rua Francisco Getúlio Vargas, 1130, Caxias do Sul, RS 95070-560, Brazil

## Abstract

Magnetoactive elastomers (MAEs) and magnetorheological elastomers (MREs) are widely explored for vibration damping, soft robotics, and biomimetic applications. Conventional ferromagnetic fillers such as iron (Fe) and its ferrimagnetic oxides (Fe_3_O_4_, Fe_2_O_3_) provide effective magnetic actuation but suffer from low corrosion resistance and limited biocompatibility. While poly­(dimethylsiloxane) (PDMS) offers excellent biocompatibility, its integration with bare Fe/Fe_3_O_4_ particles remains challenging. In this work, we present a surface-engineering strategy to overcome these limitations by synthesizing Fe_3_O_4_@Ti core–shell particles via magnetron sputtering. The titanium shell improves chemical stability and surface compatibility, enabling better dispersion and performance within the PDMS matrix for magnetoactive applications.

## Graphical abstract

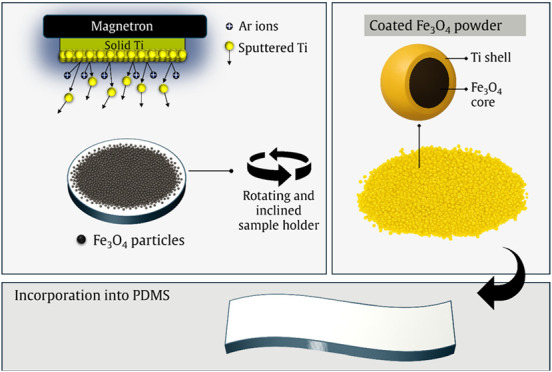

## 1 Introduction

Magnetic materials are essential for applications involving actuation, sensing, and control, with Fe and its oxides being the most employed due to their high saturation magnetization, abundance, and well-established processing methods. However, many issues arise when it is used in biological interfaces due to its limited biocompatibility.^1^

Fe plays a crucial physiological role as a cofactor in metabolic and respiratory processes, yet excessive or unbound iron can induce the formation of reactive radicals that damage DNA and cellular structures, triggering oxidative stress, inflammation, and potential carcinogenesis.^2^ Acute iron poisoning occurs in distinct clinical stages, progressing from gastrointestinal distress to systemic failure, while chronic exposure—through diet or environment—has been linked to increased cancer incidence. Although iron salts are generally safe in regulated doses, the oxidative potential of free iron remains a concern.^3,4^ Therefore, Fe needs chemical stabilization and surface modifications to ensure its safe use.^5^

To address these issues, surface functionalization of magnetic particles has been extensively investigated. Core–shell structures are promising methods for functionalizing the magnetic particles,^6–9^ as it may mitigate toxicity and improve stabilization by isolating reactive cores with more inert shells. In this context, iron oxides such as magnetite (Fe_3_O_4_) and hematite (Fe_2_O_3_) have been widely explored as cores, often coated with biocompatible materials to enhance performance in theragnostics and biomedical applications.

Beyond typical uses of iron oxides, magnetoactive and magnetorheological elastomers (MREs) have emerged as promising materials. They consist of composites with an elastomeric matrix embedded with magnetic particles, which are responsible for providing reversible deformation^10^–which enables variation of stiffness, natural frequency and damping capacity when a magnetic field is applied. As a consequence, MREs have been developed for vibration control and damping devices, soft actuators, and sensing.^11^

Magnetically responsive materials have driven innovation areas where dexterity is fundamental, such as in soft robotics, particularly in delicate manipulation, minimally invasive surgery and targeted drug delivery.^12–14^

Within this framework, the present study investigates the surface modification of Fe_3_O_4_ powder via titanium (Ti) enrichment using magnetron sputtering, a precise and eco-friendly deposition technique for creating uniform coatings. This approach provides a clean, solvent-free, and scalable surface modification route for magnetic powders, enabling shell formation without altering particle morphology or relying on wet-chemical processes. The Ti layer is expected to act as a protective and biocompatible barrier, reducing corrosion and minimizing the release of toxic iron ions. This work aims to assess the chemical, structural, and biological properties of Ti-coated Fe_3_O_4_ powders, providing insights into their potential use in magnetoactive elastomers designed for soft, biocompatible actuation, such as artificial muscles or biointerfaces.

## 2 Materials and Methods

### 2.1 Sample Preparation

Preliminary testing was carried out to study the influence of Fe_3_O_4_ fillings on mechanical strength, with 10, 20, and 30 wt % in a Silpuran PDMS (Wacker). The proportions were weighed on a 4-digit precision scale, then the samples were poured over a stainless-steel mold and cured at room temperature.

Fe_3_O_4_ particles were used in this study (see Supporting Information, Table S1, for composition analysis). Two size fractions were prepared according to ASTM E11: a coarse fraction defined as particles passing a 40-mesh sieve (≤425 μm), and a fine fraction defined as particles passing a 200-mesh sieve (≤75 μm), ensuring a consistent particle-size distribution. Scanning electron microscopy (SEM) imaging revealed an average particle size of 275 ± 60 μm for the coarse powder and 35 ± 10 μm for the fine powder (see Supporting Information, Figure S1). Poly­(dimethylsiloxane) (PDMS) composites were cured at room temperature without an external magnetic field to promote isotropic particle distribution.

In this work, the term Fe_3_O_4_@Ti denotes Fe_3_O_4_ particles coated with a Ti layer deposited by magnetron sputtering. For core–shell Fe_3_O_4_@Ti particles, physical vapor deposition was carried out via a magnetron sputtering equipment, in which the Fe_3_O_4_ filling powder was inclined and rotating at a speed of 26 rpm. This procedure ensures a uniform deposition over the powder particles. Three varying masses were enriched with titanium (15, 20, and 30 g). The parameters can be viewed in Table 1.

**Table 1:** Deposition Parameters for the Fe_3_O_4_ Powder

sample	time (min)	power (W)	sample holder rotation (rpm)	base pressure (mbar)	working pressure (mbar)
deposition over Fe_**3**_O_4_ powder					
15 g	30	100	26	5 × 10^–6^	5 × 10^–3^
20 g					
30 g					

These parameters were selected based on previous observations and sputter rate for Ti. Table 2 displays sample coding based on properties that were changed for sample production. A preliminary assessment observed the variation of Fe_3_O_4_ content and its influence on mechanical strength. Then, the following samples were selected as the optimal PDMS/Fe blends in terms of mechanical properties.

**Table 2:** Sample Coding

sample	matrix	Fe_3_O_4_/Ti content (wt %)
Mechanical Testing		
S	PDMS	0
S + Fe_3_O_4_ 10%		10/0
S + Fe_3_O_4_ 20%		20/0
S + Fe_3_O_4_ 30%		30/0
Biological Testing		
S	PDMS	0
S + coarse Fe_3_O_4_		10/0
S + fine Fe_3_O_4_		10/0
S + Fe_3_O_4_@Ti_1_		9.945/0.055
S + Fe_3_O_4_@Ti_3_		9.963/0.027

Figure 1 schematically summarizes the two-stage sample production process. In the first stage, iron oxide powder is surface modified inside a vacuum chamber via magnetron sputtering, where a solid Ti target is bombarded by argon ions, resulting in the deposition of Ti atoms onto the particle surfaces. An inclined and continuously rotating sample holder is employed to promote uniform exposure of the powder during the coating process. In the second stage, the Ti-coated Fe_3_O_4_ powder is weighed and incorporated into the PDMS matrix by vigorous mixing, followed by curing to obtain the magnetoactive elastomer composite.

**Figure 1:**
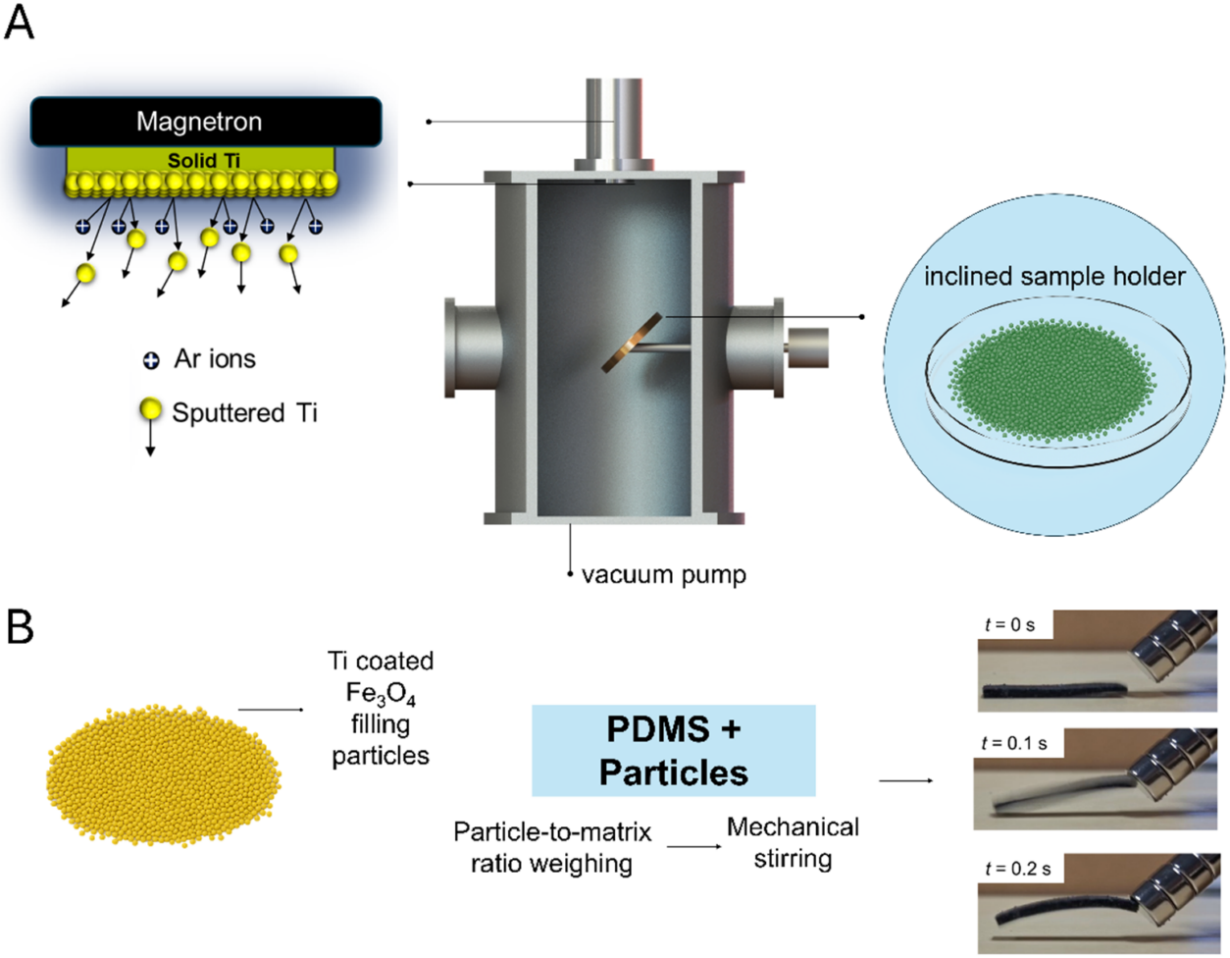
Schematic representation of the two-stage sample production process: (A) titanium coating of iron oxide powder by magnetron sputtering and (B) incorporation of Ti-coated Fe_3_O_4_ particles into the PDMS matrix to obtain a magnetoactive elastomer after curing.

### 2.2 Tensile Testing and Hardness Evaluation

Tensile testing was carried out using a universal testing machine (EMIC DL 3000) at a speed of 500 mm min^–1^, following standard ASTM D412.^15^ Hardness was measured with a digital Shore A hardness tester.

### 2.3 Chemical Analysis

Titanium deposition was quantified by X-ray fluorescence (XRF) with a Shimadzu 7000 instrument. Each powder sample was analyzed in triplicate to determine the average Ti content and the corresponding standard deviation. Additional characterization of the deposited layers was carried out by scanning electron microscopy (SEM; MIRA 3, Tescan), coupled with an Oxford Instruments X-Act silicon drift detector (SDD) for energy-dispersive X-ray spectroscopy (EDS). EDS point spectra and elemental mapping were collected from the powder samples. An additional microscope (VEGA 3, Tescan) coupled with an EDS detector (Bruker Nano, XFlash 6–10) was used for some of the micrographs.

### 2.4 Rheological Characterization

The storage modulus (*G′*) and loss modulus (*G″*) were evaluated using a rheometer (MCR-301, Anton Paar, Germany) equipped with a magneto-rheological cell (MRD-180/1T). Disc-shaped specimens (20 mm in diameter, 1 mm in thickness) were tested at a constant strain γ = 0.01% and angular frequency of ω = 10 rad s^–1^. The magnetic flux density (*B*), hereafter referred to as the applied magnetic field, varied by tuning the electric current from 5 mA up to 5 A, corresponding to a maximum *B* of ∼700 mT. Measurements were conducted at 26 ± 1 °C.

### 2.5 X-ray Diffraction (XRD)

Following hydration of Fe_3_O_4_ powders and observed corrosion, X-ray diffraction was used to evaluate the byproducts of corrosion in the particles before and after Ti coatings over the particles. The analyses were done in 2θ between 25 and 50 ° in a Cu–Kα radiation of λ = 1.5406 Å through a diffractometer (Model XRD-6000, Shimadzu, Japan).

### 2.6 Raman Spectroscopy

Raman spectra were collected on a LabRAM HR Evolution system using a 633 nm laser, 50× LWD objective and a 600 gr/mm grating. The spectra were acquired from 49–1500 cm^–1^, with 20 s acquisition time and 3 accumulations. The detector (Synapse CCD) was kept at ∼−70 °C, and a 50% ND filter was used to avoid sample heating. The spectral resolution of the setup was ∼4.7 cm^–1^.

### 2.7 Leaching Test

To evaluate iron release from the PDMS films into a liquid medium, a simulated body fluid (SBF) solution was prepared according to Kokubo’s protocol. The samples were immersed in Erlenmeyer flasks containing SBF at a ratio of 4.5 mL cm^–2^ and maintained under agitation on an orbital shaker (Novatecnica) at 150 rpm. Extracts were collected after 1, 2, and 7 days of incubation for subsequent analysis by XRF (Shimadzu 7000), which has a Fe detection threshold of 1 ppm. For this analysis, a calibration curve method was used, with FeCl_3_·6H_2_O diluted in ultrapure water (Milli-Q, MilliporeSigma) in various concentrations, ranging from 0 to 500 ppm. All tests were performed in three repetitions for two different conditions, one without Ti enrichment and the other with the highest Ti content (S + Fe_3_O_4_ 10% and S + Fe_3_O_4_@Ti_1_).

### 2.8 Biological Characterization

#### 2.8.1 Cytotoxicity Assay (MTT)

Cytotoxicity was measured through the MTT (3-(4,5-dimethylthiazol-2-yl)-2,5-diphenyltetrazolium bromide) assay method that measures integrity of the mitochondrial dehydrogenase enzyme in the formation of formazan crystals. Initially 8 × 10^5^ cells mL^–1^ L929 cells (mouse fibroblast) were seeded in 200 μL of DMEM culture medium supplemented with 10% fetal bovine serum (FBS) and 1% penicillin/streptomycin (P/S). Cells were incubated after 24 h in contact with the extraction solution obtained by immersing the samples for 24 h. DMEM medium was used for the negative control and 100 μL (1 mg·mL^–1^) of MTT was added to each well after removal of medium for 2 h. The formazan crystals were dissolved into 200 μL of DMSO (dimethyl sulfoxide) after removal of MTT solution. Reading was performed at 570 nm in a microplate reader (Spectramax me2, Molecular Devices, USA) and the results were expressed as a percentage of cell viability, with absorbance of the negative control equivalent to 100% viability and treated cells were calculated as a percentage of the control. Changes in cell viability were observed and registered after 1 day of exposure.

#### 2.8.2 Cell Adhesion

L929 cells (mouse fibroblast) were seeded in six-well plates at a density of 2 × 10^5^ cells·mL^–1^ on the samples for 1 day using 2000 μL of DMEM culture medium supplemented with 10% fetal bovine serum (FBS) and 1% penicillin/streptomycin (P/S). For fixation, cells were incubated with a 3% glutaraldehyde solution in PBS (v/v) for 15 min and dehydrated with 30, 50, 70, 90, and 100% (v/v) ethanol for 3 min at each concentration. Finally, the samples were kept in a desiccator until SEM/FEG and EDS analyses were performed.

## 3 Results and Discussion

### 3.1 Tensile Tests

The initial evaluation aimed to identify the optimal loading of Fe_3_O_4_ filler particles for incorporation into the PDMS matrix, with minimal compromise to tensile strength. Notably, the use of coarse Fe_3_O_4_ powder resulted in slightly inferior mechanical properties. Therefore, unless otherwise specified, all samples were prepared with fine-mesh Fe_3_O_4_ particles to ensure better performance.

Figure 2A illustrates the tensile strength of PDMS composites as a function of Fe_3_O_4_ content (wt %), starting from pristine Silpuran silicone (denoted as S). Remarkably, the sample with the lowest Fe_3_O_4_ loading exhibited the highest tensile strength, suggesting an optimal reinforcement effect at low particle concentration. Figure 2B shows a clear trend of increasing Shore A hardness with increasing Fe_3_O_4_ content, indicating enhanced stiffness in the silicone matrix. The observed increased hardness and decreased tensile strength along with increased particle content are consistent with previous research.^16,17^

**Figure 2:**
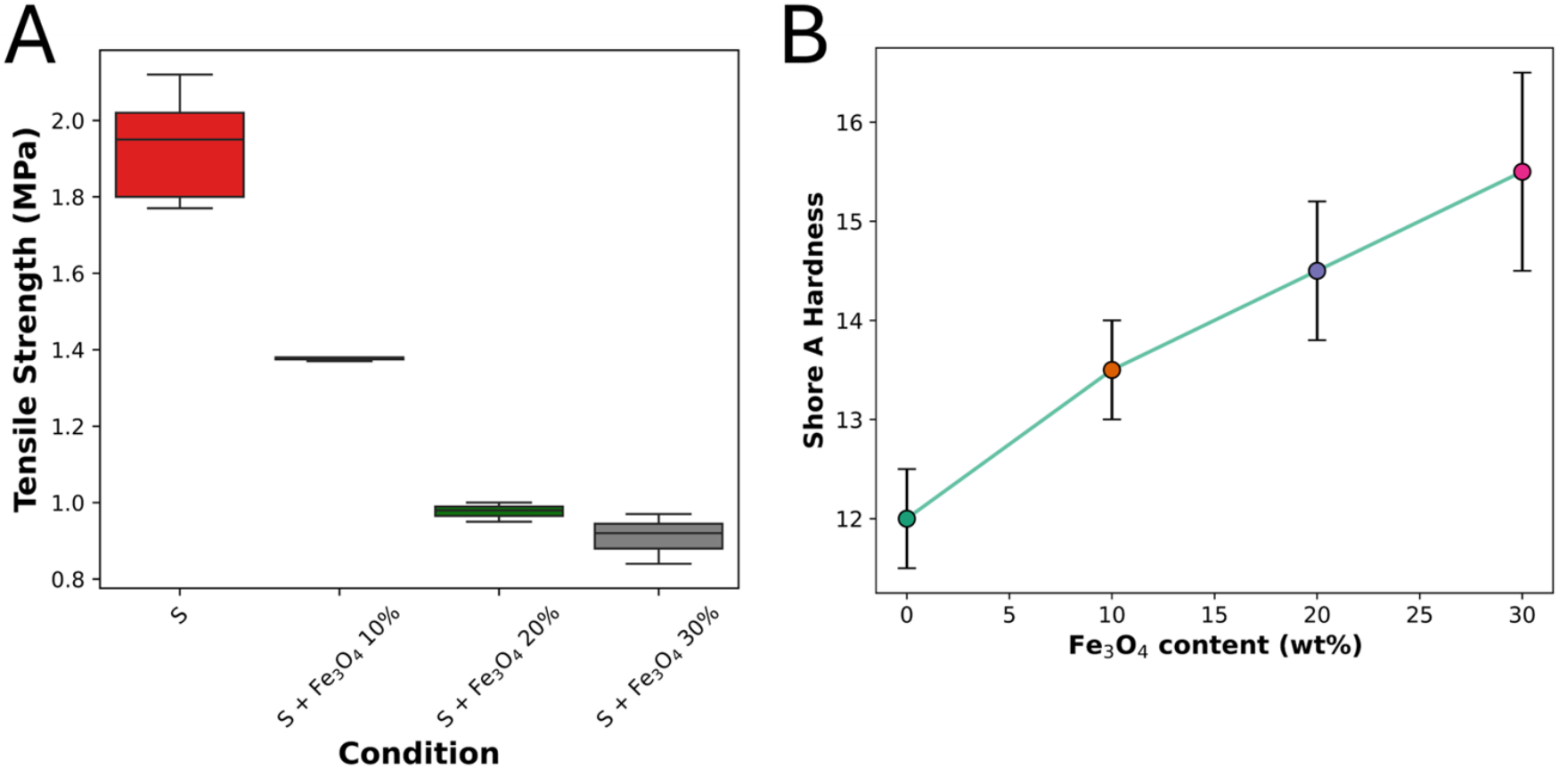
(A) Tensile strength as a function of Fe_3_O_4_ content (wt %). **S** represents Silpuran silicone. (B) Shore A hardness variation in silicone as a function of Fe_3_O_4_ content.

The increase in Fe_3_O_4_ content promotes the formation of stress concentrations and discontinuities in the PDMS matrix, as observed in SEM images (see Supporting Information, Figure S2), which leads to surface wrinkling and cracking. These morphological changes compromise the structural integrity of the composite, which is reflected in the reduction of its mechanical properties, as evidenced by the tensile tests. Additionally, fine powder provides better mechanical properties in comparison to coarser powder. Miscibility concerns are especially true due to the mismatch in properties and nonuniformity in particle distribution. A progressive decline in elongation at break (%) was also observed with increasing Fe_3_O_4_ content, with reductions of 16.37%, 11.24%, and 8.91% corresponding to each 10 wt % increment in iron oxide concentration.

### 3.2 Chemical Analysis of Ti Depositions

Ti content was measured under three different conditions: 15, 20, and 30 g. These samples were coated under identical sputtering conditions, with only the mass in sample holder being changed. The results obtained by XRF are displayed in Figure 3. Less powder weight ends up containing more Ti in mass fraction compared to samples with higher contents, 15 g of Fe_3_O_4_ powder result in a Ti content of 0.55 ± 0.02%. The Ti weight content for the 20 and 30 g samples falls within the experimental error (0.30 ± 0.02% and 0.27 ± 0.03%), indicating that increasing powder mass reduces differences in Ti mass fraction.

**Figure 3:**
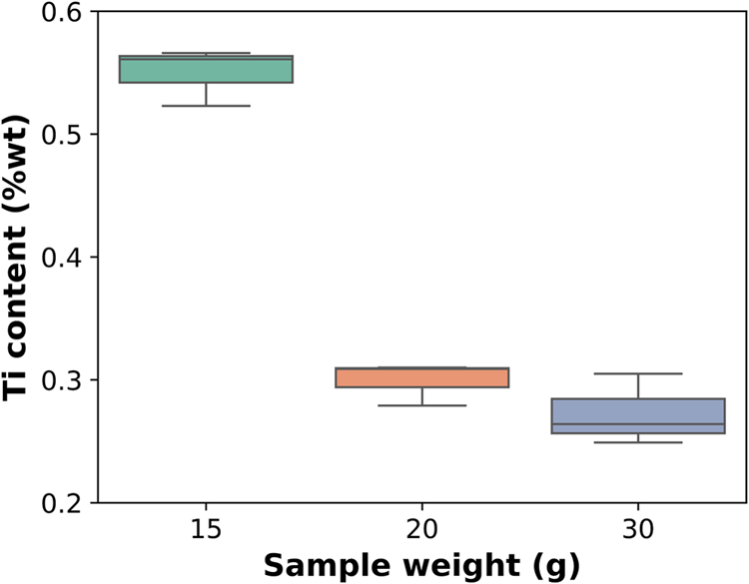
Ti content after magnetron sputtering carried out under identical conditions as a function of sample weight.

These results further corroborate that magnetron sputtering is a technique which enables microdosing and even NP synthesis when the proper parameters are employed.^18–20^

EDS surface mapping and point spectra were used to assess the coating of the particles. Figure 4 presents a scanning electron microscopy micrograph of a single Fe_3_O_4_@Ti_1_ particle. On the left side of the image, EDS elemental maps for Fe and Ti illustrate their spatial distribution across the particle. On the right side, a point spectrum reveals characteristic peaks corresponding to both elements, confirming their presence. It is important to note that EDS analysis can penetrate to depths of several micrometers, which explains the detection of iron beneath the titanium-rich surface layer.

**Figure 4:**
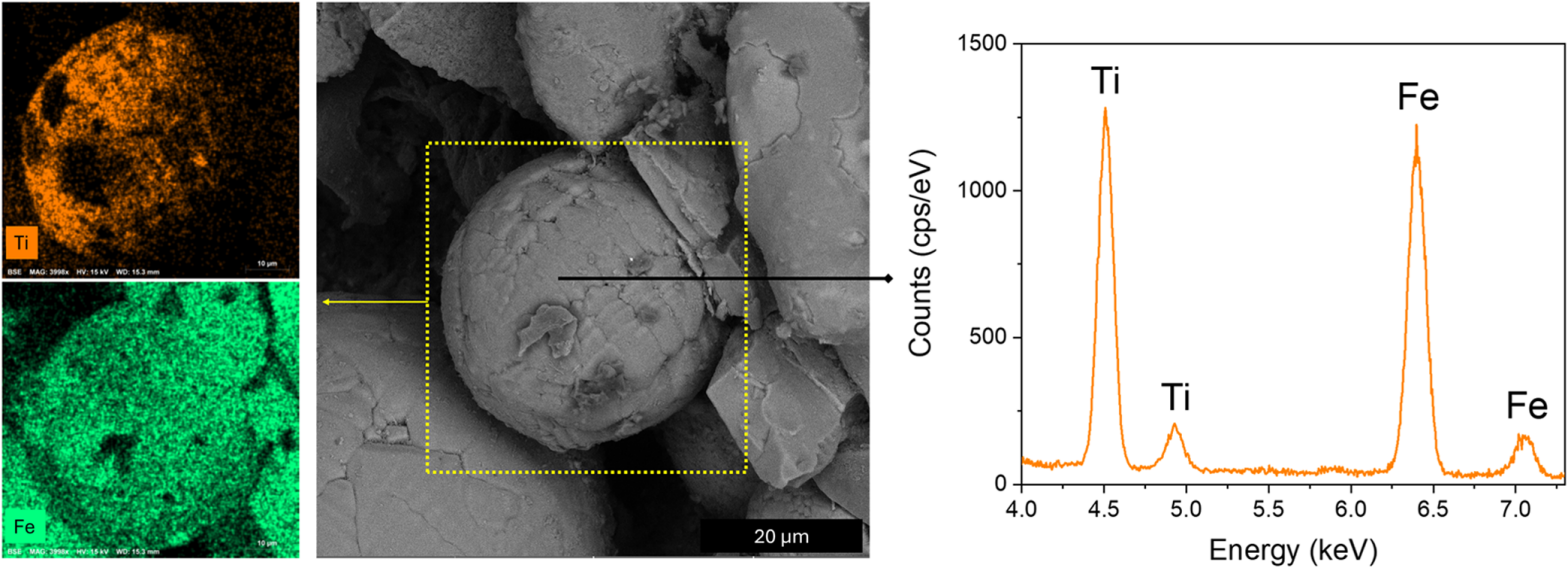
SEM micrograph illustrating the surface coating of a single Fe_3_O_4_@Ti_1_ particle. *Left:* EDS elemental maps reveal the spatial distribution of Fe and Ti across the particle. *Right:* EDS point spectrum highlights the characteristic peaks corresponding to Fe and Ti.

The Ti layer thickness (∼250 nm) was estimated from the sputtering rate and deposition time. It should be noted that this value is an approximation, as no direct thickness measurements were performed. EDS analysis confirms the presence of Ti on the particle surface but does not allow accurate determination of coating thickness or uniformity. For further characterization, Figure S3 (Supporting Information) presents EDS point spectra of Fe_3_O_4_@Ti_3_, revealing a weaker Ti signal compared to Fe_3_O_4_@Ti_1_.

### 3.3 Leached Iron

PDMS is widely regarded as an effective host matrix, primarily due to its chemical inertness and its ability to retain embedded particles with minimal dispersion or loss.^21,22^ Nonetheless, iron leaching was assessed via XRF. The amount of leached iron from the films is shown in Figure 5.

**Figure 5:**
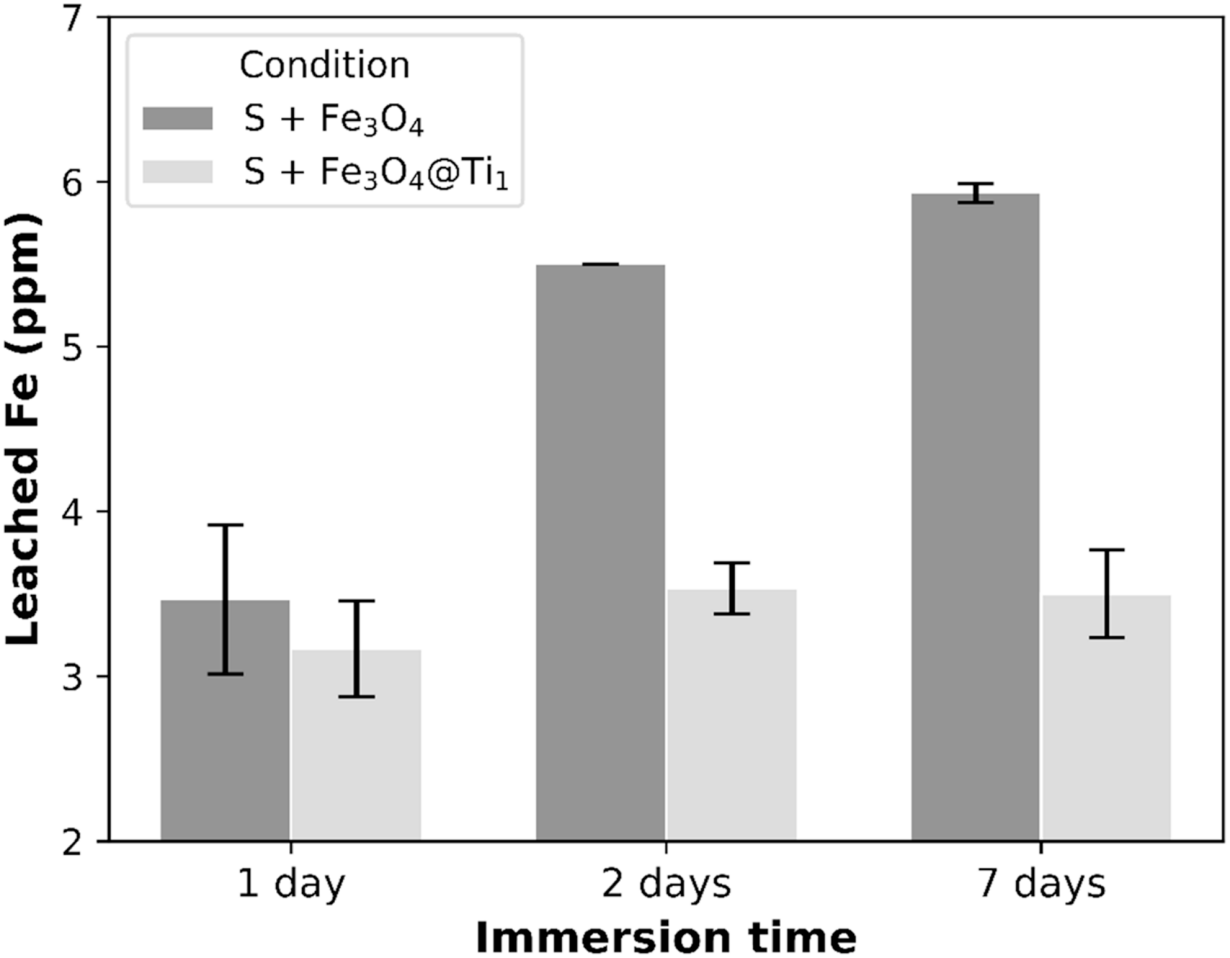
Leached Fe in SBF at different immersion times for S + Fe_3_O_4_ and S + Fe_3_O_4_@Ti1 conditions. Error bars correspond to the standard deviation of three replicates.

The amount of iron leached raises concerns, especially due to its poor corrosion resistance. In biological environments, this issue is especially important, as both temperature and reactive media accelerate corrosion processes. Given that the literature indicates iron concentrations above 5.6 mg L^–1^ will start to impair HepG2 cell viability *in vitro*,^23^ while dozens of mg L^–1^ cause genotoxic and cytotoxic effects. Limits between 50 and 75 ppm are suggested elsewhere.^24,25^ In this case, the amount is negligible and is unlikely to cause any harm to cells. This is particularly evident for PDMS containing Ti-coated Fe_3_O_4_ powder, where a leached Fe threshold appears to exist below 4 ppm. Moreover, the amount of leached Fe remains remarkably stable over time compared to that of bare Fe_3_O_4_ particles.

### 3.4 Dynamic Mechanical Properties under Magnetic Field

Ferrimagnetic Fe_3_O_4_ possesses field-alignable magnetic moments capable of generating magnetostrictive strain. This strain can modify local stress fields within the composite, as demonstrated in magnetoelastic polymer–ferrite systems.^26^ A similar mechanism likely contributes to the stress redistribution observed in our Fe_3_O_4_-containing samples.

Although the PDMS composites clearly exhibit static magnetic behavior, as evidenced by attraction to a permanent magnet, no measurable magnetorheological response was detected up to an applied magnetic field of 700 mT, as observed in Figure 6. This experiment was intended as a validation of composite processability and magnetic filler incorporation, rather than a demonstration of optimized magnetorheological performance. This invariance of *G*′, *G″*, and tan δ indicates that the particle fraction and dispersion within the matrix are insufficient to form field-induced chain-like structures, which are necessary to modify the viscoelastic response. The low volumetric content of magnetic particles (∼1.3 vol %) combined with the stiffness of the PDMS matrix likely prevents particle mobility and reorientation under the applied field. Therefore, while the composites are magnetically active, they do not exhibit a significant magnetorheological effect under the tested conditions. Differences between the formulations were observed, with PDMS containing bare Fe_3_O_4_ exhibiting the highest storage and loss moduli, while pristine PDMS showed the lowest values.

**Figure 6:**
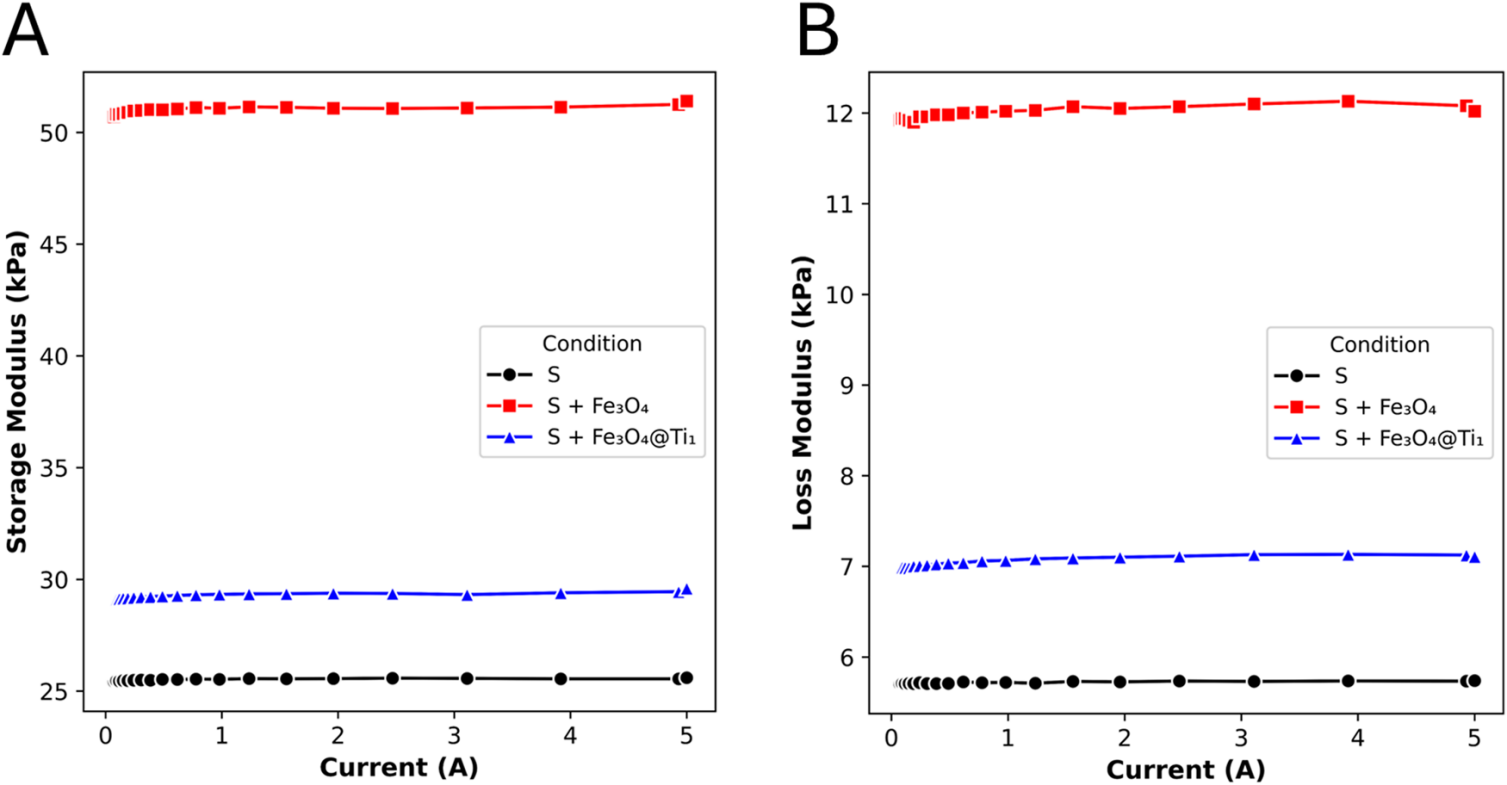
Storage, *G*′ (A), and loss, *G*″ (B), moduli for the neat PDMS (S), as well as for PDMS containing Fe_3_O_4_ fillers (S + Fe_3_O_4_) and Ti-coated Fe_3_O_4_ fillers (S + Fe_3_O_4_@Ti_1_) as a function of applied current.

Typically, magnetorheological elastomers reported in the literature employ magnetic particle contents ranging from 30 to 90 wt %,^27–30^ although the biological and mechanical properties are often not investigated or fully characterized, limiting the assessment of their potential for biomedical applications. Overall, these results emphasize the need to carefully balance particle loading, matrix properties, and biocompatibility in the design of MRE-based biomaterials when high strain or pronounced field-responsive behavior is desired. Using a softer matrix, a higher particle content, as well as anisotropic curing, could be varied for observation of significant magnetorheological effects.

### 3.5 X-ray Diffraction (XRD) and Raman Spectroscopy

Different Fe_3_O_4_ powders were hydrated and dried in a desiccator. After 7 days, samples were observed to see signs of oxidation. Visibly, rust appeared more pronounced in samples without Ti coating, but also in Fe_3_O_4_@Ti_3_ – the sample with the least amount of Ti over Fe. Fe_3_O_4_@Ti_1_ did not show visible rust (see Supporting Information, Figure S4).

To assess the extent of corrosion in the samples, X-ray Diffraction (XRD) analysis was performed. Figure 7 presents the XRD patterns of the investigated samples. Oxidized fine Fe_3_O_4_ powder revealed an additional reflection beyond those of α-Fe and Fe_3_O_4_. The peak at 31.7° can correspond to an intermediate hydrated iron oxide phase formed during the hydration–drying cycle, such as poorly crystalline FeOOH or mixed Fe^2+^/Fe^3+^ oxyhydroxides. For this reason, Raman spectra were also assessed. The small peak near 42° could be attributed to FeO. The scarcity of distinct matching peaks, coupled with potential spectral overlaps, limits definitive phase identification. Amorphous or nanocrystalline rust phases (e.g., ferrihydrite) may be present below the XRD detection limit. In contrast, Fe_3_O_4_@Ti_1_ retained a metallic gray appearance, and its diffractograms were indistinguishable from bare Fe_3_O_4_, indicating the absence of detectable crystalline corrosion products under the tested conditions.

**Figure 7:**
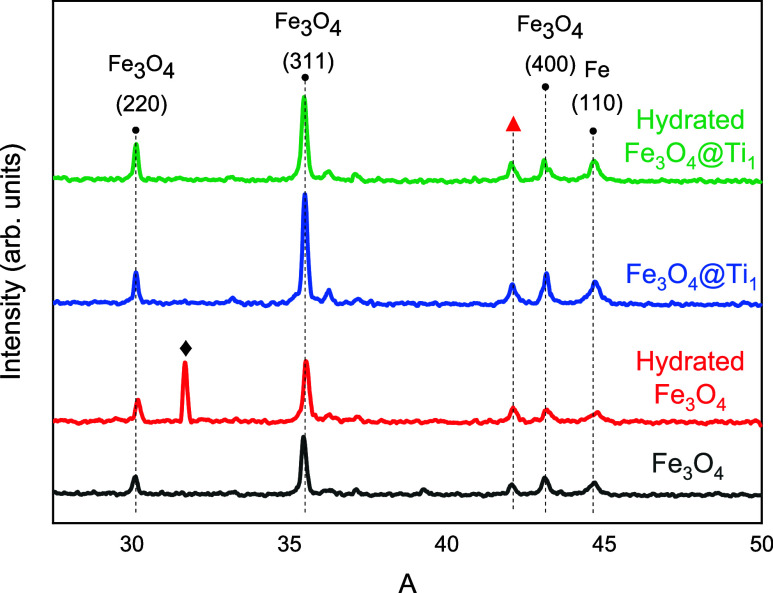
XRD patterns for samples Fe_3_O4 and Fe_3_O_4_@Ti_1_ before and after hydration and drying.

Crystallographic Information Files (CIF) from Crystallography Open Database were simulated using VESTA software (version 3.9.5a). The following CIF codes were used: 1100108 (metallic Fe) and 1010369 (Fe_3_O_4_). No significant strains were observed for the peaks in the XRD patterns.

The Raman spectrum of pristine Fe_3_O_4_ shown in Figure 8 displays the characteristic modes of both hematite (∼224 cm^–1^, 290 cm^–1^, 408 cm^–1^, 611 cm^–1^, and 1318 cm^–1^) and magnetite (660 cm^–1^). This indicates partial surface oxidation of the particles.

**Figure 8:**
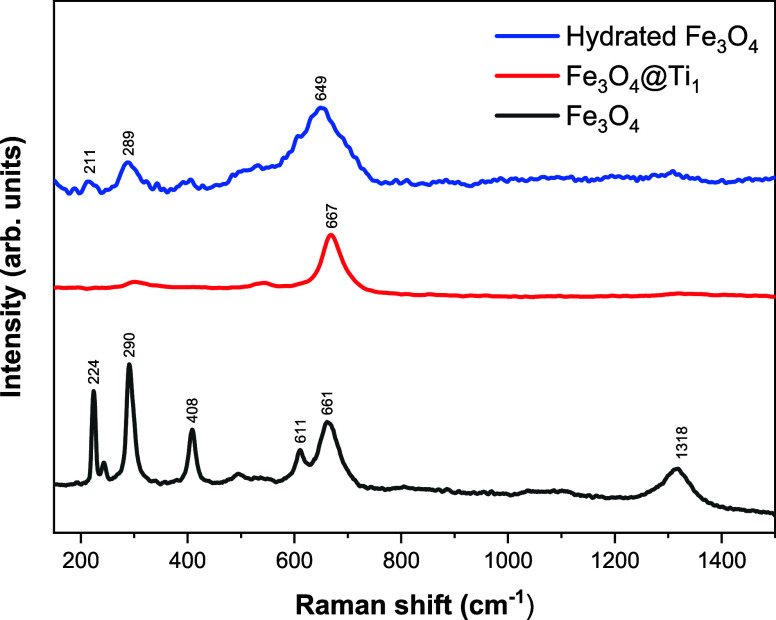
Raman spectra for different Fe_3_O_4_ conditions, including neat condition, Ti-coated and hydrated and dried sample.

With Ti deposited particles (Fe_3_O_4_@Ti_1_), these features vanish almost completely, and a single intense band emerges at ∼667 cm^–1^, consistent with magnetite. This attenuation of the Fe_3_O_4_ Raman modes suggests that the Ti-based layer effectively delays oxidation. In contrast, hydrated Fe_3_O_4_ displays a shifted broad band at ∼649 cm^–1^, characteristic of disordered, hydroxyl-rich iron oxide surfaces. Overall, the spectrum resembles that of corrosion products reported elsewhere.^31^ No bands characteristic of TiO_2_ (anatase or rutile) were detected.

### 3.6 Biological Characterization

MTT assays were used to evaluate biocompatibility in L929 cells (Figure 9). According to ISO standards, all samples are considered nontoxic, since all groups presented cell viability over 70%. Pristine sample (S) showed the highest proliferation rate for cells, while Fe_3_O_4_-containing samples showed a slight reduction in cell viability. This aligns well with the biocompatible nature of PDMS and the low amount of leached iron, which in great concentration could contribute to a limited cell viability.

**Figure 9:**
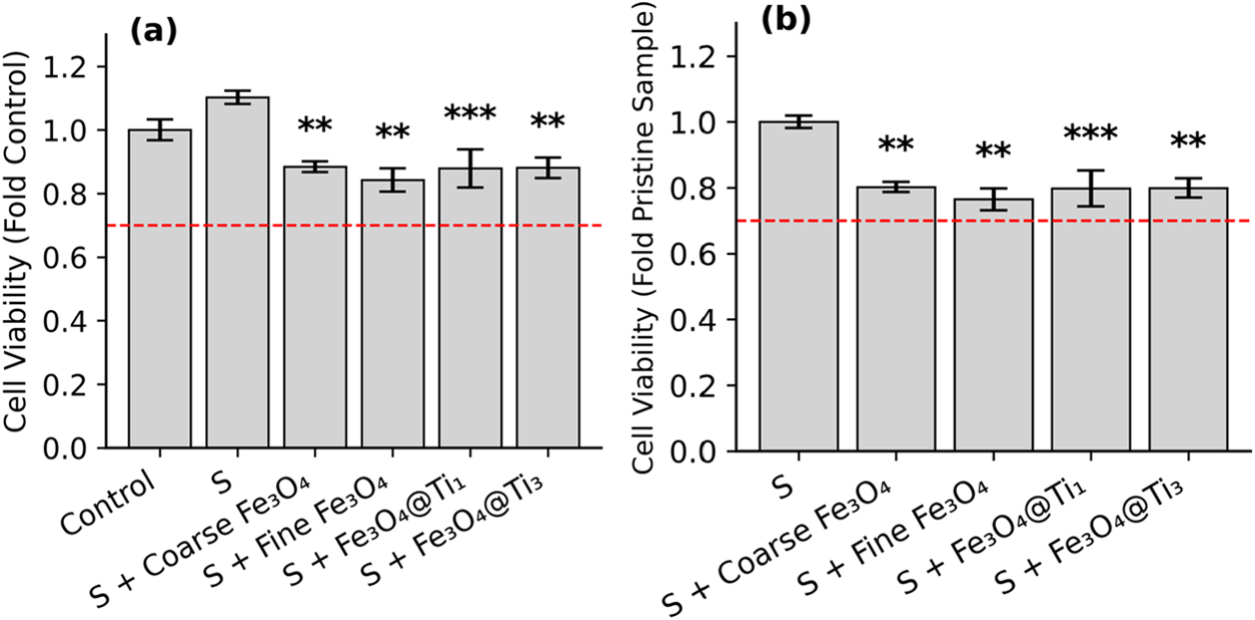
Cell viability of samples normalized to different baselines. (a) Cell viability of treated samples expressed as fold change relative to the untreated control. The pristine sample (S) shows increased viability, while all iron-containing samples reduce cell viability to varying degrees. (b) Cell viability recalculated using the pristine sample (S) as the normalization reference, highlighting the impact of Fe-based incorporation. Dashed red line indicates the viability threshold of 0.7. Asterisks represent statistical significance relative to the reference sample (*p* < 0.01 (**), p < 0.001 (***)) based on Tukey HSD test following one-way ANOVA.

L929 cell adhesion was assessed under different conditions through SEM imaging (Figure 10). For the pristine PDMS condition, cells displayed a spindle-like morphology with elongated cytoplasmic extensions, which is a strong indication of anchorage and favorable early adhesion. Conversely, for all the other conditions, cells appeared predominantly round, suggesting a reduced ability to spread throughout the surfaces. This trend indicates that the incorporation of both coated and uncoated Fe_3_O_4_ particles weakens initial cell–substrate interaction. This agrees with reduced interfacial compatibility and alterations in surface stiffness arising from particle–matrix interactions.

**Figure 10:**
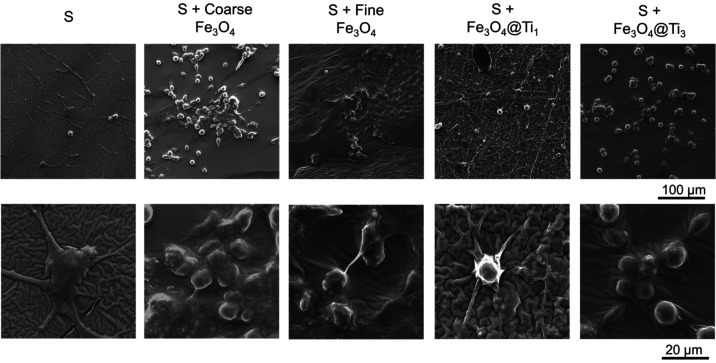
SEM micrographs showing cell adhesion in two different magnifications. Scale bars shown in the lower right of each row apply to all images in the corresponding row.

Another notable trend was the higher concentration of cells around wrinkled or irregular regions. This suggests that the surface features are partially responsible for providing anchoring sites for cell adhesion, which is a common observation in works addressing surface morphological effects on cells.

From these observations, all samples exhibited limited extracellular matrix spreading and anchorage compared with the pristine condition. For facilitating the visualization of cell adhesion, EDS concentration maps were employed. The maps for Si and C are presented in Figure 11 for given conditions.

**Figure 11:**
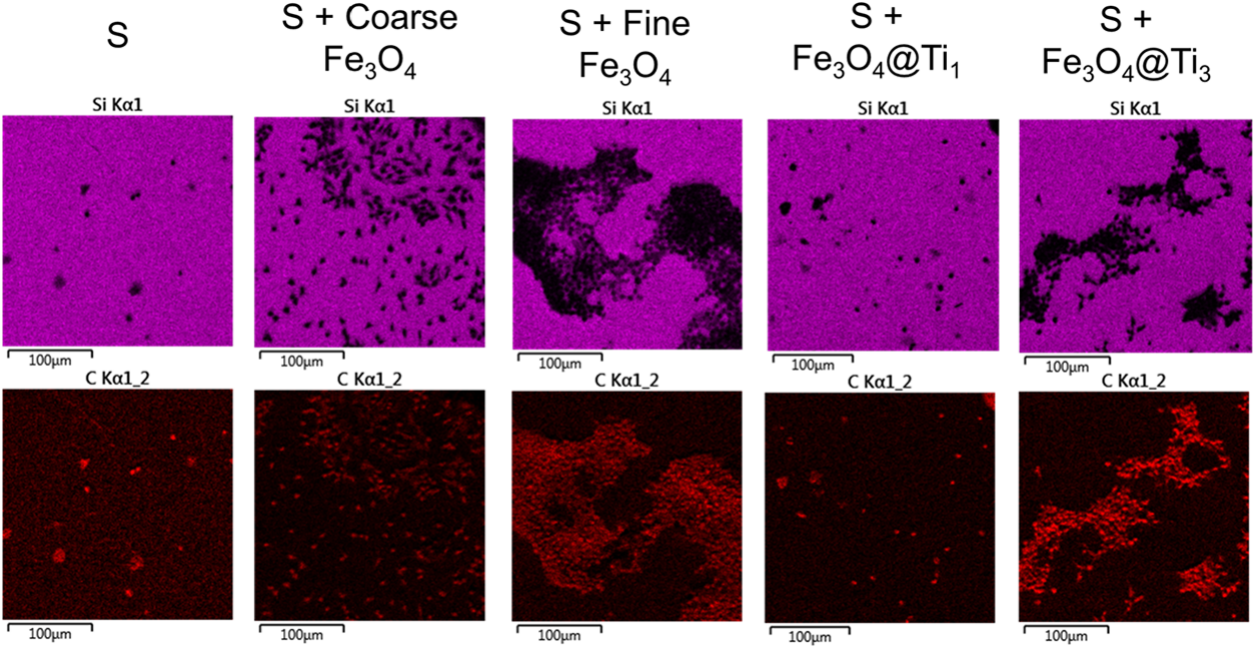
EDS concentration maps for Si and C showing cell distribution over the different substrates.

The maps helped distinguish surface and cell regions for a clear visualization of adhesion patterns. Cell clusters were evident in various conditions. In Figure 11, these clusters are observed in the carbon concentration maps for S + fine Fe_3_O_4_ and S + Fe_3_O_4_@Ti_3_. The clustering behavior is further evidence that while the composites can support cell adhesion, the interaction is weaker and more spatially discontinuous compared to pristine PDMS.

Overall, Figures 9 and 10 show that particle incorporation influences interfacial bonding and alters initial cell adhesion. Surface morphology appears to mitigate such effects. This indicates that there is a combined effect of chemical composition, substrate stiffness and topographical changes which modulate cell interactions. These *in vitro* assays are limited to short-term cytocompatibility and do not account for long-term or *in vivo* biological responses.

## 4 Conclusion

Magnetron sputtering is a versatile and eco-friendly physical vapor deposition technique, and its adaptation for coating powder materials offers a novel pathway for the fabrication of core–shell structures. In this study, titanium was deposited onto iron oxide particles using a custom-designed, inclined rotating chamber, which promotes continuous powder movement and enables uniform coating. Three key findings emerged:

(1) The amount of powder within the chamber had a significant effect on coating uniformity, suggesting that the process is scalable while preserving deposition quality. This opens opportunities for tailoring nanoparticle properties through precise control of sputtering parameters.
(2) The titanium coating notably improved the corrosion resistance of the Fe_3_O_4_ particles, demonstrating that surface modification can effectively tune material properties with minimal additional mass.
(3) For magnetoactive applications, particularly those requiring compatibility with biological environments, the use of coated particles presents a promising strategy to improve biocompatibility and toxicity limitations of bare ferromagnetic materials.

Furthermore, as expected, size and coating influence performance of iron oxide particles. This should be prioritized to understand magnetorheological properties as well as biocompatibility. According to literature reports, core–shell Fe_3_O_4_ structures often exhibit a significant reduction in saturation magnetization due to the presence of a nonmagnetic shell^32,33^ and it is expected that the Ti coating has the same effect on the particles. and a similar effect is expected for the Ti coating used in this work. Direct magnetic characterization was not included in the present study; future investigations will be required to confirm the expected reduction in magnetic response due to the Ti shell.

Future investigations may also explore PDMS matrices enriched with higher weight fractions of coated Fe_3_O_4_ particles for a more pronounced magnetorheological effect, as well as to assess the behavior of Fe_3_O_4_@Ti particles under cyclic magnetic stimulation for potential fatigue effects. Additionally, alternative coatings could be applied to tailor the surface chemistry of magnetic particles for specific applications, particularly to delay corrosion onset, including evaluations under both basic and acidic environments. Coating thickness is another critical parameter that warrants systematic investigation, as it may significantly influence saturation magnetization and other physicochemical properties.

Finally, *in vitro* biocompatibility studies conducted under magnetic field stimulation could provide valuable insights into how stiffness and magnetically active domains modulate cellular adhesion dynamics. Comparative assessments using softer and stiffer elastomers may further clarify the interactions between modified magnetic particles and the surrounding polymeric matrix.

## Supplementary Material



## Data Availability

All data supporting the findings of this study are included in the manuscript and the Supporting Information. Additional data are available from the corresponding author upon reasonable request.

## Notes

Additional morphological and compositional characterization: FEG-SEM particle size analysis, SEM observations of surface wrinkling induced by different particulate additives, SEM/EDS evaluation of titanium coating on Fe_3_O_4_ particles, qualitative assessment of hydration–drying effects, and XRF chemical composition analysis of Fe_3_O_4_ particles (PDF)
