# Supplementary material for: Synthesis of Iron(II,III) Oxide–Titanium Core–Shell Particles via Magnetron Sputtering for Magnetoactive Elastomers
Source: ACS Omega. 2026 Mar 3;11(10):16034–42. doi: 10.1021/acsomega.5c10485 (PMC13000593; doi:10.1021/acsomega.5c10485)
Supplement: Supplementary file 1 [file ao5c10485_si_001.pdf]

Supporting Information

Synthesis of Iron(II,III) Oxide–Titanium Core–Shell Particles via Magnetron Sputtering for Magneto-Active Elastomers

Cristian Padilha Fontoura[1], Amanda Poletto Santi[2], Wellington Vieira de Souza[1], Mariana Roesch Ely[2], Cesar Aguzzoli[1]

[1] Área do Conhecimento de Ciências Exatas e Engenharias, Graduate Program in Materials Science (PPGMAT), Universidade de Caxias do Sul, Rua Francisco Getúlio Vargas, 1130, Caxias do Sul, RS 95070-560, Brazil

[2] Instituto de Biotecnologia, Universidade de Caxias do Sul, Rua Francisco Getúlio Vargas, 1130, Caxias do Sul, RS 95070-560, Brazil

Table of Contents

Supporting Information Figures and Tables

1. **Figure S1**. FEG-SEM micrographs display particle size for (a) fine Fe3O4 powder and (b) coarse Fe3O4 powder. 2
2. **Figure S2**. FEG-SEM micrographs illustrating surface wrinkling patterns and cracks induced by the incorporation of three different additives: Fe_3O_4, SiO_2 and ZrO_2. The images reveal distinct morphological differences, highlighting the influence of each additive on surface texture and surface deformation. 2
3. **Figure S3**. SEM micrograph and EDS spectra illustrating the presence of Ti in Fe_3O_4@Ti_3 particles. 3
4. **Figure S4**. Visual appearance of samples 7 days after hydration and subsequent drying. Surface changes and color variations reflect the effects of water exposure and drying over time 3
5. **Table S1**. Chemical composition of Fe3O4 filling 3

Particle size was quantified by measuring the diameters of ten individual Fe_3O_4 particles from FEG-SEM micrographs. The coarse powder exhibited an average particle size of 275 ± 60 µm, whereas the fine powder showed an average of 35 ± 10 µm. Representative examples of these measurements are provided in **Figure S1**.

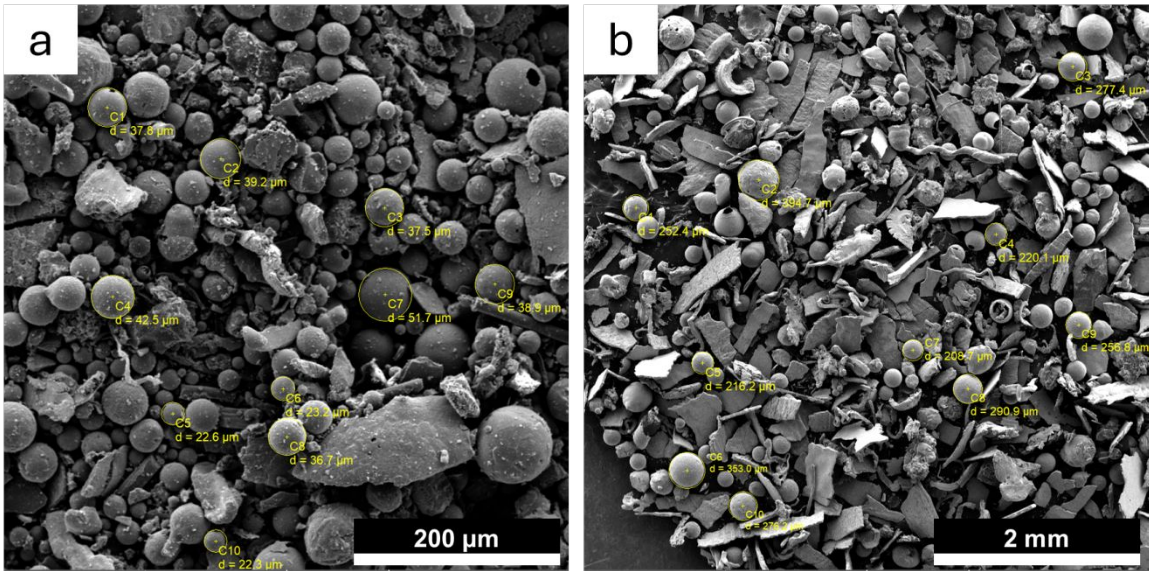

Figure S1. FEG-SEM micrographs display particle size for (a) fine Fe_3O_4 powder and (b) coarse Fe_3O_4 powder.

Addition of other powder-form particles also result in wrinkling of PDMS, which was observed for the cases of SiO_2 and ZrO_2. This is, therefore, not a feature only inherent to Fe_3O_4 filling addition, viewed in **Figure S2**.

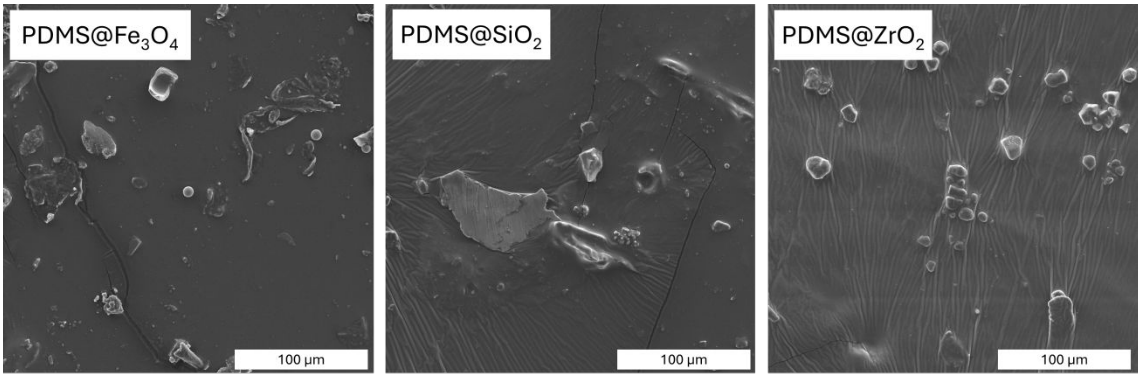

Figure S2. FEG-SEM micrographs illustrating surface wrinkling patterns and cracks induced by the incorporation of three different additives: Fe_3O_4, SiO_2 and ZrO_2. The images reveal distinct morphological differences, highlighting the influence of each additive on surface texture and surface deformation.

Elemental analysis via SEM/EDS revealed distinct features of titanium coverage on iron powder. As shown in **Figure S3**, the Fe_3O_4@Ti_3 sample exhibits a noticeably lower titanium content compared to other formulations. In contrast to Fe_3O_4@Ti_1, titanium detection in Fe_3O_4@Ti_3 is significantly reduced, with an approximate weight percentage detected via XRF nearly half that observed in Fe_3O_4@Ti_1. A subtle shoulder observed in the spectrum near the Ti-Kα suggest the small amount of Ti.

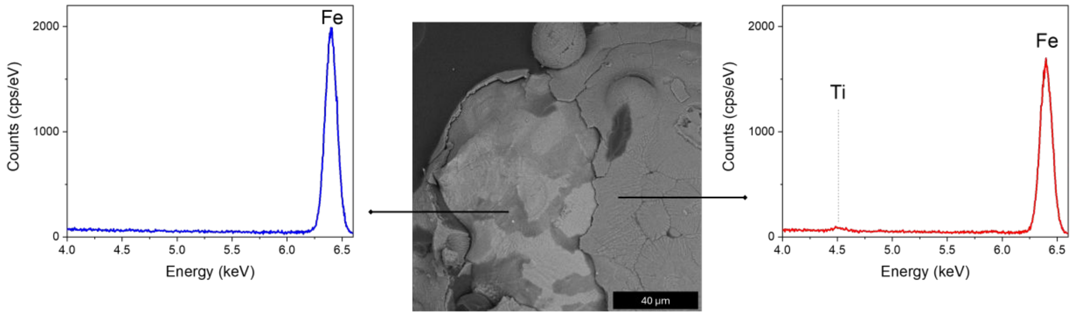

Figure S3. SEM micrograph and EDS spectra illustrating the presence of Ti in Fe_3O_4@Ti_3 particles.

Hydration and drying of different Fe_3O_4 powders were qualitatively analyzed before XRD analysis. **Figure S4** displays how the corrosion onset is visualized in these samples.

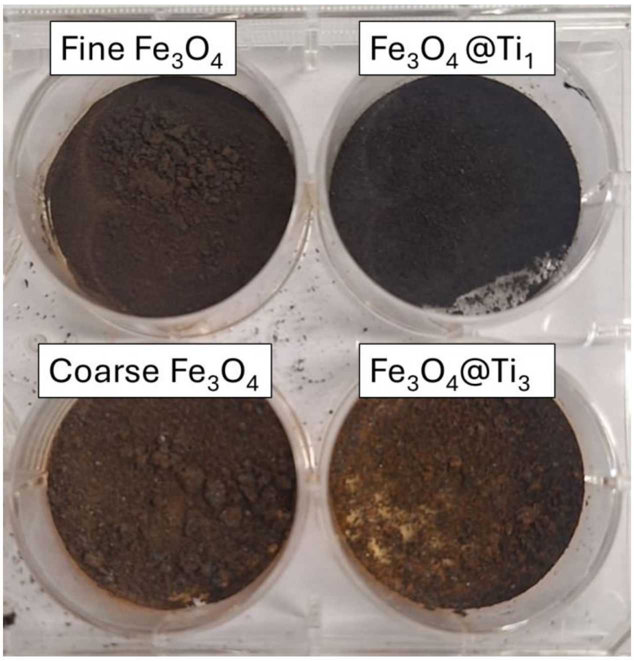

Figure S4. Visual appearance of samples 7 days after hydration and subsequent drying. Surface changes and color variations reflect the effects of water exposure and drying over time.

Fe_3O_4 fillings were analyzed through XRF. Note: Carbon and oxygen are not detected via XRF. The average results are presented in **Table S1**.

Table S1. Chemical composition of Fe_3O_4 filling

**Element**	**Mass fraction (%)**
Fe	89.514 ± 0.12
Si	3.513 ± 0.06
Al	2.505 ± 0.10
Ca	1.195 ± 0.01
S	0.726 ± 0.01
Others	2.547 ± 0.01
